# Fatal Case of Diarrhœa

**Published:** 1816-10

**Authors:** John Bailey

**Affiliations:** Surgeon


### *FATAL CASE of DIARRHŒA,*

By John Bailey, Surgeon.

THE following Case, unattractive from its simplicity, shews how feebly the attachments of our mortal nature may be connected; and conveys an useful lesson to Practitioners, to be guarded in expressing their opinion, even upon the most trite and common occurrences.

Hezekiah Hipwell, a lad of 17 or 18 years of age, on Monday the 10th of June, 1816, considered himself in perfect health. On the following day, after eating a hearty dinner, he became sick and vomited: next day he had a diarrhœa, but without any pain or continuance of vomiting. He resisted the advice of those about him to call in medical aid, considering, as he was exempt from pain, that there could not be any occasion for it. On the succeeding day, (Thursday) his friends requested me to see him; he had had frequent purgation through the night, and appeared from that cause alone to be excessively faint; he was not sick at the stomach, but free from all ventral pain; the only uneasiness that he made mention of was in his wrists, a symptom not easily to be accounted for, as having any connection with a diarrhœa. I ordered for him twenty minims of tinct. opii in a little aq. menthæ; and at intervals, warm Port wine spiced with cloves. This was about two in the afternoon. In the evening I revisited him; he had kept down the draught with a little broth, but he refused to take any thing further from a seeming difficulty of swallowing. His head was thrown back, and the pulsation at the wrist so feeble as scarcely to be felt; the extremities were parting with their heat. At three in the morning, without any suffering, and without any more than a very common and usually harmless ailment, he took his leave of this world. His abrupt departure out of this life, and the unaccountable cause for it, were strong inducements for an inspection of the body. Leave was granted for that purpose; and in the evening of Friday, the day on which he died, I proceeded, accompanied by two assistants, and one of your learned Editors, to the examination. I need not go through the detail of individual visceral appearances, there being little or no alteration in any of them. The only parts that shewed any diseased alteration were the internal coat of the stomach and the ileum; the former was more vascular than common, and exhibited proofs of inflammation in a slight degree. The latter also was triflingly affected in a similar way. I have seen, on examining dead bodies, such appearances, where there was, during life, no symptom of intestinal disorder.

The record of a case like this, although it holds up to view none of those fanciful aberrations of Nature, which garnish some anatomical reports, is nevertheless worthy of notice, inasmuch as it admonishes every judicious practitioner of our art, to be cautious and hesitating in the delivery of his opinion. Who would have conceived that a mild diarrhœa, of less than two days' continuance, should of itself have brought on dissolution. The existence of some other disease, organic lesion, or the administration of poison, had not dissection proved the contrary, might, with some reason, have given colour to such an unusual event. On reporting the appearances of this young man's dissection to his friends, I learned, for the first time, that the pain which he complained of in his wrists arose from considerable exertion in playing cricket on the evening of Monday, the day before he was taken unwell; hence, it seems, that that uneasiness was unconnected with the bowel disorder.

*Harwich, August 2,* 1816.

PART

